# Preoperative Hemoglobin Level, Oxygen Saturation and Postoperative Outcomes in Children With Cyanotic Congenital Heart Disease: A Propensity-Score Matching Analysis

**DOI:** 10.3389/fped.2021.762241

**Published:** 2022-01-21

**Authors:** Dan Zhou, Li-Jing Deng, Yun-Fei Ling, Meng-Lin Tang

**Affiliations:** ^1^Pediatric Intensive Care Unit, West China Hospital, Sichuan University, Chengdu, China; ^2^West China School of Nursing, Sichuan University, Chengdu, China; ^3^Department of Cardiac Surgery, West China Hospital, Sichuan University, Chengdu, China

**Keywords:** children, cyanosis, hemoglobin, oxygen saturation, congenital heart disease, outcome

## Abstract

**Background:**

The optimal preoperative hemoglobin (Hb) level is difficult to define in children with cyanotic congenital heart disease (CHD) due to hypoxemia-induced secondary erythrocytosis. This retrospective study integrated preoperative Hb and pulse oxygen saturation (SpO_2_) using the product of Hb × SpO_2_ to predict postoperative outcomes in children with cyanotic CHD.

**Patients and Methods:**

Children aged <18 years undergoing cardiac surgery with cyanotic CHD were included. The cutoff value of Hb × SpO_2_ was the age-adjusted lower limit of normal Hb (aaHb) in healthy children. The main outcomes were in-hospital death and the composite outcome of severe postoperative events. Multivariate logistic regression analysis and propensity score matching analysis were used to adjust for important confounders.

**Results:**

The presence of preoperative Hb × SpO_2_ < aaHb was observed in 21.6% of cyanotic children (*n* = 777). Children with Hb × SpO_2_ < aaHb had higher in-hospital mortality (12.5% vs. 4.6%, *P* < 0.001) and composite outcome incidence (69.6% vs. 32.3%, *P* < 0.001) than those with Hb × SpO_2_ ≥ aaHb. After propensity score matching, 141 pairs of children were successfully matched. Multivariate analysis showed that preoperative Hb × SpO_2_ < aaHb was significantly associated with the composite outcome in the entire population (odds ratio = 4.092, 95% confidence interval = 2.748–6.095, *P* < 0.001) and the matched cohorts (odds ratio = 2.277, 95% confidence interval = 1.366–3.795, *P* = 0.002).

**Conclusion:**

Our results suggest that a preoperative Hb × SpO_2_ value below the lower limit of normal hemoglobin is a prognostic factor in cyanotic children undergoing cardiac surgery and is a potential criterion to evaluate preoperative anemia in this population.

## Introduction

Anemia is a common disease in pediatrics, presenting in 15–74% of this population (1–6). A reduction in oxygen-carrying capacity can lead to inadequate tissue oxygenation and organ function (7). As a result, anemia may impair the body's resistance to surgical stress. Recent studies indicate that preoperative anemia is associated with higher in-hospital mortality in children undergoing noncardiac surgery (2, 3), but little is known about this association in children undergoing cardiac surgery. Limited evidence has shown that preoperative hemoglobin (Hb) <11.0 g/dl is associated with a higher risk of postoperative acute kidney injury in children with congenital heart disease (CHD) (8).

CHD can be divided into two categories according to the presence or absence of cyanosis (9–11). Cyanosis and hypoxemia may occur if a significant right-to-left shunt exists in a heart defect, and generally, these are defects with significant complexity (10). Cyanosis can increase early and late postoperative mortality in children undergoing cardiac surgery (9) and enhance the long-term risks of noncardiac surgery and type 2 diabetes mellitus in adulthood (10, 11). Although cyanotic CHD is more complex and associated with poor clinical outcomes, cyanotic patients were excluded in some anemia-related studies (6, 7). The main obstacle is that the optimal Hb level is difficult in cyanotic patients when hypoxemia leads to secondary erythrocytosis and an increase in the Hb concentration (12, 13).

The main function of hemoglobin is to transport oxygen. Under normal nutritional and hematopoietic conditions, there is an inverse relationship between the severity of compensatory erythrocytosis and resting oxygen saturation in cyanotic patients (14). Therefore, this study integrated preoperative Hb and oxygen saturation to predict postoperative outcomes in children with cyanotic CHD.

## Patient and Methods

### Patient Population and Data Collection

This retrospective study was conducted in a university-affiliated tertiary hospital using data from December 2008 to December 2018. Children were included if (1) they were younger than 18 years; (2) their diagnosis was CHD; (3) their heart defects led to right-to-left shunts, cyanosis and hypoxemia (resting SpO_2_ ≤ 90%); and (4) they underwent open heart surgery. Children were excluded if (1) their preoperative Hb and resting SpO_2_ were unavailable; (2) they had preoperative invasive or noninvasive mechanical ventilation; (3) they received preoperative blood transfusion; or (4) they had diseases that resulted in abnormal Hb quality, such as thalassemia and carbon monoxide poisoning.

### Data Collection and Definition

The following clinical data were collected: age, sex, disease history, altitude of long-term residence, clubbed fingers or toes, preoperative comorbidities, routine blood analysis results, resting SpO_2_, echocardiographic record, type of heart defect, Risk Adjustment for Congenital Heart Surgery 1 (RACHS-1) score (15), duration of invasive mechanical ventilation, and postoperative adverse events. Preoperative comorbidities within 1 month prior to surgery included abnormal liver and kidney function, stroke, thyroid disease, respiratory disease, heart disease and infection. The resting SpO_2_ data without oxygen therapy within 2 weeks prior to surgery measured by the noninvasive pulse oximeter were recorded, and the average SpO_2_ was calculated for children with multiple measurements of different fingers and toes. Echocardiographic data included left ventricular ejection fraction, pulmonary arterial hypertension, patent ductus arteriosus and aortopulmonary collateral arteries. For patients with a single ventricle, the ejection fraction of the single ventricle was recorded.

Postoperative children were followed up until discharge or in-hospital death. A severe adverse event is any unfortunate occurrence that either results in death or a life-threatening event prolonging the length of hospital stay (16), including in-hospital death, cardiac arrest, sepsis, reoperation, severe hemorrhage, and the duration of invasive mechanical ventilation in 75–100th percentile. The main outcomes were in-hospital death and the composite of severe postoperative adverse events. Sepsis in this study refers to conditions previously termed *severe sepsis* in the international pediatric sepsis consensus (17). Severe hemorrhage included intracranial hemorrhage, acute bleeding-induced hemodynamic instability and a 20% decrease in Hb levels.

### Mathematical Model for Integrating Hemoglobin and Oxygen Saturation

Physically dissolved oxygen is rare, and noninvasive SpO_2_ could replace invasive arterial oxygen saturation (SaO_2_) tests (18), so the arterial oxygen content (CaO_2_) for blood with normal Hb quality could be estimated according to the formula:


$$\begin{array}{l} \text{Ca}{\text{O}}_{2}\left(\text{ml}/\text{dl}\right)=1.39\left(\text{ml}/\text{g}\right)\times \text{Hb}\left(\text{g}/\text{dl}\right)\times \text{Sp}{\text{O}}_{2}\left(\text{\%}\right)÷100\;\left(\text{a}\right) \end{array}$$

An adaptive response to chronic hypoxemia was to enhance erythropoiesis, and the increase in erythrocytes is inversely related to resting oxygen saturation in patients with sufficient iron stores and an appropriate erythropoietic response (14). The estimation formula of CaO_2_ also supports this inverse relationship. Therefore, we presumed that CaO_2_ could assess whether patients had adequate secondary erythrocytosis. If cyanotic children had sufficient compensation for preoperative Hb concentration, they could obtain a similar CaO_2_ to healthy children:


$$\begin{array}{l} 1.39\times \text{Preoperative Hb }\times \text{ preoperative Sp}O_{2}=1.39\\ \;\begin{array}{r} \begin{array}{l} \times \text{Normal Hb}\times \text{normal Sp}O_{2}\;(b) \end{array} \end{array} \end{array}$$

$$\begin{array}{l} \text{Preoperative Hb}\times {\text{preoperativeSpO}}_{2}=\text{Normal Hb}\\ \;\times \text{normal Sp}O_{2}\;(c) \end{array}$$

The age-adjusted lower limits of normal hemoglobin (aaHb) in healthy children are 14.5 g/dl for neonates, 9 g/dl at 2 months, 10.5 g/dl at 6 months, 11.5 g/dl at 2 years, and 12 g/dl and 13 g/dl in adolescent girls and boys, respectively (1, 19). Therefore, the formula can be converted as follows:


$$\begin{array}{l} \text{Preoperative Hb}\times {\text{preoperativeSpO}}_{2}\geq \text{aaHb}\times \text{normal Sp}O_{2}(d) \end{array}$$

In contrast, the presence of preoperative Hb × SpO_2_ < aaHb × normal SpO_2_ indicated that CaO_2_ in cyanotic children was lower than that in healthy children, with insufficient compensation for preoperative Hb concentration. The values of normal SpO_2_ range from 95 to 100%.

### Statistical Analysis

Continuous variables are reported as the mean ± standard deviation or median (range). Categorical variables are presented as frequency counts and percentages and were analyzed using the Chi-squared test. If appropriate, Fisher's exact tests were performed. The optimal cutoff value of the continuous variables used to predict the composite outcome was calculated using Youden's index after performing receiver operating characteristic curve analysis. In the propensity-score matching analysis, the presence or absence of preoperative Hb × SpO_2_ < aaHb × normal SpO_2_ was entered in the model as a dependent variable, and covariates that should be statistically (*P* < 0.100 in the Chi-squared test) associated with the composite outcome were included in the model as independent variables. They were preoperative SpO_2_, age, RACHS-1, type of heart defect, clubbed fingers or toes, preoperative comorbidity, patent ductus arteriosus, pulmonary arterial hypertension and aortopulmonary collateral arteries. The matching ratio was 1:1, and the matching tolerance was 0.001. Before multivariate analysis, collinearity was diagnosed. If the variance proportion >0.5 existed in two or more covariates in the same dimension or a variance inflation factor (VIF) was up to 3, the presence of collinearity was considered. Multivariate analysis was performed using the backward stepwise method of logistic regression. All statistical tests were performed using SPSS v.24 (IBM Corp., Armonk, NY), and a two-sided *P* < 0.05 indicated a statistically significant difference.

## Results

### Characteristics of Children

In total, 805 children met the inclusion criteria, and 28 cases were excluded: 22 children had preoperative invasive or noninvasive mechanical ventilation, preoperative Hb or resting SpO_2_ was unavailable in 4 cases, and 2 children received preoperative blood transfusions. Among the remaining 777 eligible children, the median age was 24.7 (range 0–214) months, with 359 girls and 418 boys. The top 5 heart defects were tetralogy of Fallot (39.3%), double-outlet right ventricle (16.4%), single ventricle (8.3%), pulmonary atresia (8.1%), and transposition of great arteries (7.4%). The remaining defect types included hypoplastic right heart syndrome, total anomalous pulmonary vein drainage, hypoplastic left heart syndrome, complete atrioventricular septal defect, interrupted aortic arch, truncus arteriosus and other rare defects. In-hospital mortality was 6.3%, and the incidence of the composite outcome was 40.4%.

### The Distribution and Linear Relation of Preoperative Hb and Resting SpO_2_ in Cyanotic Children

**Figure 1:**
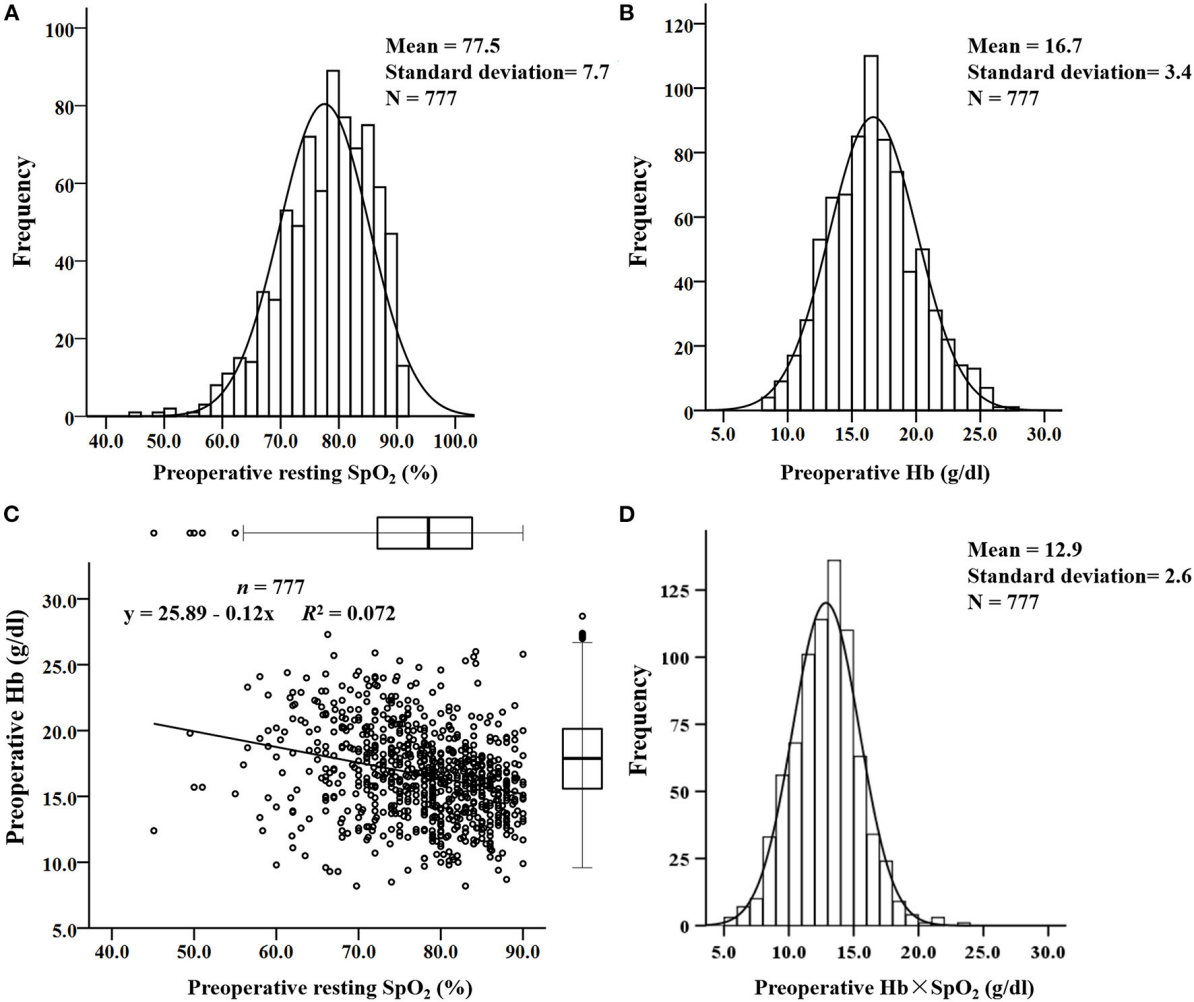
The distribution and linear relationship of resting pulse oxygen saturation (SpO_2_) and preoperative hemoglobin (Hb) concentration in cyanotic children. **(A)** Distribution of resting SpO_2_; **(B)** distribution of preoperative Hb concentration; **(C)** linear relationship between preoperative Hb concentration and resting SpO_2_; **(D)** distribution of the preoperative Hb × SpO_2_ value.

Preoperative resting SpO_2_ without oxygen therapy and preoperative Hb concentration were in accordance with normal distributions (Figures 1A,B). The mean values were 77.5% ±7.7% and 16.7 ± 3.4 g/dl, respectively. The linear relation analysis showed a poor correlation between preoperative Hb and SpO_2_ (*R*^2^ = 0.072, Figure 1C). The mean value of preoperative Hb × SpO_2_ was 12.9 ± 2.6 g/dl and a normal distribution was also shown (Figure 1D). To predict the composite outcome, Youden's index showed that 100% was the optimal cutoff value in the range of normal SpO_2_ (95% to 100%) to evaluate whether cyanotic children had sufficient compensation for preoperative Hb concentration.

### Association of Preoperative Hb Level and Postoperative Mortality and Morbidity

The rates of preoperative Hb < aaHb and preoperative Hb × SpO_2_ < aaHb were 4.5% and 21.6%, respectively. Both of them were significantly associated with the composite outcome (Table 1), but preoperative Hb × SpO_2_ < aaHb was associated with more types of severe postoperative events, especially associated with higher in-hospital mortality (12.5% vs. 4.6%, *P* < 0.001, Table 1).

**Table 1:** Association of preoperative hemoglobin levels with postoperative outcomes in cyanotic children.

**Variable**	**Hb < aaHb** **(***n =*** 742)**	**Hb ≥ aaHb** **(***n =*** 35)**	***P*** **-value**	**Hb × SpO_**2**_ < aaHb** **(***n =*** 609)**	**Hb × SpO_**2**_ ≥ aaHb** **(***n =*** 168)**	***P*** **-value**
Reoperation	31 (4.2%)	1 (2.9%)	>0.999^a^	22 (3.6%)	10 (6.0%)	0.177
Severe hemorrhage	43 (5.8%)	2 (5.7%)	>0.999^a^	29 (4.8%)	16 (9.5%)	0.019
In-hospital death	45 (6.1%)	4 (11.4%)	0.270^a^	28 (4.6%)	21 (12.5%)	< 0.001
Cardiac arrest	65 (8.8%)	7 (20.0%)	0.035^a^	40 (6.6%)	32 (19.0%)	< 0.001
Invasive ventilation ≥168 h^b^	166 (22.4%)	18 (51.4%)	<0.001	121 (19.9%)	63 (37.5%)	< 0.001
Sepsis	207 (27.9%)	21 (60.0%)	<0.001	141 (23.2%)	87 (51.8%)	< 0.001
Composite outcome	286 (38.5%)	28 (80.0%)	<0.001	197 (32.3%)	117 (69.6%)	< 0.001

a *Fisher's exact test*.

b *The 75th percentile of invasive ventilation duration was 167 h. aaHb, age-adjusted lower limit of normal hemoglobin in healthy children; Hb, hemoglobin; SpO_2_, pulse oxygen saturation*.

### Risk Factors for Postoperative Composite Outcome and In-hospital Death

In addition to the preoperative Hb level, Chi-squared tests showed that age at the cutoff value of 2 years, RACHS-1 at the cutoff value of 3 scores, type of heart defect, clubbed fingers or toes, preoperative comorbidity and patent ductus arteriosus were significantly (*P* < 0.05) associated with the postoperative composite outcome, and SpO_2_ at the cutoff value of 74.1% (*P* = 0.084), pulmonary arterial hypertension (*P* = 0.092) and aortopulmonary collateral arteries (*P* = 0.095) were slightly associated with the composite outcome. Meanwhile, preoperative comorbidity, patent ductus arteriosus, type of heart defect and RACHS-1 showed statistical associations with in-hospital death at the level of *P* < 0.05, and age and pulmonary arterial hypertension were slightly related to in-hospital death with a *P-*value of 0.050-0.100. Other factors were not associated with the composite outcome or in-hospital death in the univariate analyses.

We found that clinical factors were significantly associated with preoperative Hb × SpO_2_ < aaHb (Table 2). Therefore, collinearity diagnostics was performed. The results showed that the VIF values ranged from 1.061 to 1.300, and a variance proportion >0.5 was not observed in two or more covariates in the same dimension. Therefore, the collinearity was poor, and these covariates were suitable for multivariate analysis. Subsequently, multivariable logistic regression analysis was carried out using the backward stepwise method and showed that the presence of preoperative Hb × SpO_2_ < aaHb was associated with increased risks of in-hospital death [odds ratio (*OR*) = 2.149; 95% confidence interval (*CI*) = 1.147–4.027, *P* = 0.017] and the composite outcome (*OR* = 4.092, 95% *CI* = 2.748–6.095, *P* < 0.001) in the entire population (Table 3).

**Table 2:** Confounding factors associated with the preoperative hemoglobin level in cyanotic children.

**Variable**	**All children (***N =*** 777)**			**Matched children (***N =*** 282)**		
	**Hb × SpO_**2**_ < aaHb** **(***n =*** 609)**	**Hb × SpO_**2**_ ≥ aaHb** **(***n =*** 168)**	***P*** **-value**	**Hb × SpO_**2**_ < aaHb** **(***n =*** 141)**	**Hb × SpO_**2**_ ≥ aaHb** **(***n =*** 141)**	***P*** **-value**
**SpO**_**2**_			< 0.001			0.632
74.1–90%	439 (72.1%)	93 (55.4%)		76 (53.9%)	80 (56.7%)	
<74.1%^a^	170 (27.9%)	75 (44.6%)		65 (46.1%)	61 (43.3%)	
**Age**			< 0.001			0.323
<2 y^a^	271 (44.5%)	110 (65.5%)		93 (66.0%)	85 (60.3%)	
2y – <18 y	338 (55.5%)	58 (34.5%)		48 (34.0%)	56 (39.7%)	
**RACHS-1**			< 0.001			0.404
1–2	339 (55.7%)	67 (39.9%)		71 (50.4%)	64 (45.4%)	
3–6	270 (44.3%)	101 (60.1%)		70 (49.6%)	77 (54.6%)	
**Type of heart defect**			< 0.001			0.981
Tetralogy of Fallot	249 (40.9%)	56 (33.3%)		57 (40.4%)	52 (36.9%)	
Double-outlet right ventricle	99 (16.3%)	29 (17.3%)		29 (20.6%)	28 (19.9%)	
Single ventricle	51 (8.4%)	14 (8.3%)		13 (9.2%)	14 (9.9%)	
Pulmonary atresia	56 (9.2%)	7 (4.2%)		5 (3.5%)	7 (5.0%)	
Transposition of great arteries	31 (5.1%)	27 (16.1%)		9 (6.4%)	10 (7.1%)	
Others	123 (20.2%)	35 (20.8%)		28 (19.9%)	30 (21.3%)	
**Clubbed fingers or toes**			< 0.001			0.461
No	281 (46.1%)	115 (68.5%)		85 (60.3%)	91 (64.5%)	
Yes	328 (53.9%)	53 (31.5%)		56 (39.7%)	50 (35.5%)	
**Preoperative comorbidity**			0.001			0.776
No	590 (96.9%)	153 (91.1%)		134 (95.0%)	135 (95.7%)	
Yes	19 (3.1%)	15 (8.9%)		7 (5.0%)	6 (4.3%)	
**Aortopulmonary collateral arteries**			0.028			0.382
No	364 (59.8%)	116 (69.0%)		88 (62.4%)	95 (67.4%)	
Yes	245 (40.2%)	52 (31.0%)		53 (37.6%)	46 (32.6%)	
**Patent ductus arteriosus**			0.045			> 0.999
No	440 (72.2%)	108 (64.3%)		98 (69.5%)	98 (69.5%)	
Yes	169 (27.8%)	60 (35.7%)		43 (30.5%)	43 (30.5%)	
**Pulmonary arterial hypertension**			0.053			0.721
No	559 (91.8%)	146 (86.9%)		122 (86.5%)	124 (87.9%)	
Yes	50 (8.2%)	22 (13.1%)		19 (13.5%)	17 (12.1%)	
**Residence altitude**			0.055			0.378
<1,500 m	553 (90.8%)	144 (85.7%)		125 (88.7%)	120 (85.1%)	
≥1,500 m	56 (9.2%)	24 (14.3%)		16 (11.3%)	21 (14.9%)	
**Gender**			0.247			0.471
Boy	321 (52.7%)	97 (57.7%)		83 (58.9%)	77 (54.6%)	
Girl	288 (47.3%)	71 (42.3%)		58 (41.1%)	64 (45.4%)	
**Cardiac surgery history**			0.516			0.164
No	552 (90.6%)	155 (92.3%)		134 (95.0%)	128 (90.8%)	
Yes	57 (9.4%)	13 (7.7%)		7 (5.0%)	13 (9.2%)	
**Left ventricular ejection fraction** ^**b**^			>0.999^c^			>0.999^c^
<55%	595 (97.7%)	165 (98.2%)		138 (97.9%)	138 (97.9%)	
≥55%	14 (2.3%)	3 (1.8%)		3 (2.1%)	3 (2.1%)	

a *The optimal cutoff value to predict the composite outcome*.

b *For children with a single ventricle, the ejection fraction of the single ventricle was recorded*.

c *Fisher's exact test. aaHb, age-adjusted lower limit of normal hemoglobin in healthy children; Hb, hemoglobin; RACHS-1, the Risk Adjustment for Congenital Heart Surgery 1; SpO_2_, pulse oxygen saturation*.

**Table 3:** Multivariable analysis for the postoperative outcomes.

**Outcome**	**Variable**	**All patients (*****N*** **= 779)**		**Matched patients (*****N*** **= 282)**	
		***OR*** **(95% ***CI***)**	***P*** **-value**	***OR*** **(95% ***CI***)**	***P*** **-value**
**In-hospital death**					
RACHS-1 ≥ 3	3.258 (1.557–6.816)	0.002	3.917 (1.418–10.816)	0.008	
Patent ductus arteriosus	2.256 (1.216–4.184)	0.010	3.270 (1.424–7.512)	0.005	
Preoperative comorbidity	3.023 (1.234–7.406)	0.016	–	–	
Preoperative Hb × SpO_2_ < aaHb	2.149 (1.147–4.027)	0.017	–	–	
**Composite outcome**					
Preoperative Hb × SpO_2_ < aaHb	4.092 (2.748–6.095)	<0.001	2.277 (1.366–3.795)	0.002	
Age ≥ 2 y	0.403 (0.289–0.562)	<0.001	0.385 (0.227–0.651)	<0.001	
RACHS-1 ≥ 3	2.248 (1.616–3.127)	<0.001	2.455 (1.476–4.085)	0.001	
Aortopulmonary collateral arteries	1.686 (1.210–2.348)	0.002	–	–	
Patent ductus arteriosus	1.687 (1.184–2.404)	0.004	–	–	
Preoperative comorbidity	2.673 (1.088–6.566)	0.032	–	–	

*aaHb, age-adjusted lower limit of normal hemoglobin; CI, confidence interval; Hb, hemoglobin; OR, odds ratio; RACHS-1, the Risk Adjustment for Congenital Heart Surgery 1; SpO_2_, pulse oxygen saturation*.

As confounding factors may still influence the statistical results despite multivariate analysis, we further conducted a propensity-score matching analysis. We successfully matched 141 children with preoperative Hb × SpO_2_ < aaHb to 141 children with preoperative Hb × SpO_2_ ≥ aaHb. All confounders were balanced in the matched cohorts (Table 2). Although preoperative Hb × SpO_2_ < aaHb was not associated with in-hospital death, multivariable logistic regression analysis confirmed that preoperative Hb × SpO_2_ < aaHb was still an independent prognostic factor for the composite outcome (*OR* = 2.277, 95% *CI* = 1.366–3.795, *P* = 0.002) in the matched cohorts (Table 3).

## Discussion

Optimal preoperative Hb level and anemia criteria are difficult to define in cyanotic children (12, 13). The 15 g/dl cutoff value was adopted in the study published by Okoromah et al. (5). This value was significantly higher than the lower limits of normal Hb ranges of all age groups of healthy children (1, 19). However, a fixed Hb cutoff value may not be appropriate for cyanotic children because the severity of compensatory erythrocytosis is not fixed but inversely correlated with resting SpO_2_ (14). In cyanotic adults with compensatory erythrocytosis, a strong linear correlation was found between Hb and SpO_2_ (Hb = 61 – SpO_2_/2) (20). In addition to oxygen saturation, age may also influence the Hb level of children (1, 19). In children with cyanotic heart disease, the regression equation was found as follows: Hb concentratio*n* = 34.4 – 0.22 × (aortic oxygen saturation) + 0.14 × age (21). However, this regression equation was restricted to children with sufficient iron stores and oxygen saturation >75% (21). Based on the theory of an inverse relationship between the severity of compensatory erythrocytosis and resting oxygen saturation (14) and the estimation formula of CaO_2_ (18), we presumed that the value of preoperative Hb × SpO_2_ can evaluate whether cyanotic children achieved adequate Hb compensation. If the value of preoperative Hb × SpO_2_ of cyanotic children was below the lower limit of normal Hb of each age group, this indicated that the preoperative Hb concentration was not sufficient to obtain similar CaO_2_ to healthy children, and preoperative anemia would be considered.

Preoperative anemia is common in neonates and children undergoing noncardiac operations, with an estimated incidence of 24–32% (2, 3). Similarly, the incidence was 23% in acyanotic children with a ventricular septal defect or an atrioventricular canal (4). However, the rate of preoperative anemia was only 4.5% in our cyanotic children if the diagnosis of anemia was made based on the presence of actual Hb concentration < aaHb. Hypoxemia may induce secondary erythrocytosis and then increase the hemoglobin concentration of cyanotic patients (12, 13). Obviously, the incidence of preoperative anemia in cyanotic children would be seriously underestimated by the actual Hb concentration, but the anemia rate could increase to 21.6% according to the Hb × SpO_2_ value, which may improve the detection rate of preoperative anemia in cyanotic children. Moreover, univariate analyses indicated that the presence of preoperative Hb × SpO_2_ < aaHb was associated with more types of severe postoperative events, including in-hospital mortality (Table 1). Both multivariable logistic regression analysis and propensity-score matching analysis showed that preoperative Hb × SpO_2_ < aaHb was significantly associated with the composite postoperative outcome (Table 3). This was consistent with the findings that preoperative anemia was associated with poor postoperative outcomes in adults undergoing cardiac surgery (7, 22, 23) and in neonates and children undergoing noncardiac surgery (2, 3). Therefore, compared with the actual Hb concentration, the value of Hb × SpO_2_ below the lower limit of normal Hb of each age group has stronger prognostic power and may be more suitable to evaluate preoperative anemia in children with cyanotic CHD.

The reasons for poor prognosis among anemic patients undergoing surgery are not well known. One explanation is that anemia may be the representation of other confounding factors related to poor prognosis (7, 13). Erythrocytosis does not tend to stabilize until hypoxemia has been present for quite some time, so preoperative Hb × SpO_2_ < aaHb was more likely to occur in children without adequate time for the response to hypoxemia, demonstrated by our results that the rates of preoperative Hb × SpO_2_ < aaHb in children younger than 6 months (33.7%) and aged 6 months to 2 years (27.1%) were higher than those in other age groups (14.2–17.2%). Meanwhile, younger age was an independent risk factor for the postoperative composite outcome (Table 3), which was consistent with the finding in acute kidney injury after congenital cardiac surgery (8). We also found that the incidences of Hb × SpO_2_ < aaHb were higher in children with patent ductus arteriosus (26.2%) or transposition of great arteries (46.6%) and were lower in children with aortopulmonary collateral arteries (17.5%) or clubbed fingers or toes (13.9%). Compared to the entire population with a median age of 24.7 months, the median age was younger in children with PDA (13.5 months) or transposition of great arteries (10.3 months) and was older in children with aortopulmonary collateral arteries (31.8 months) or clubbed fingers or toes (48.9 months). Children with transposition of great arteries or duct-dependent CHD usually require early diagnosis and intervention (24, 25) and may not have enough time to compensate for hemoglobin. In contrast, the presence of aortopulmonary collateral arteries and clubbed fingers and toes generally indicates that hypoxemia has existed for a long time. Therefore, anatomic and pathophysiologic factors may partially explain the prognostic power of Hb × SpO_2_ < aaHb. A previous study revealed that aortic oxygen saturation below 75–80% was associated with an increase in erythropoietin titer, suggesting that adequate and stable erythrocytic response is not easy for patients with deep hypoxemia (21, 26). Similarly, our findings showed that low SpO_2_ (<74.1%) was associated with insufficient compensation for preoperative Hb concentration (Hb × SpO_2_ < aaHb) (Table 2). In addition to cyanotic CHD, living at high altitudes (low oxygen environment) can also lead to elevated hemoglobin levels in patients. In this study, eighty (10.3%) patients lived at an altitude of >1,500 m. However, the altitude of long-term residence was only slightly related to the presence of Hb × SpO_2_ < aaHb (*P* = 0.055), probably because children moving from high altitude to the urban area of Chengdu with a 500-m altitude had adapted to the normal oxygen environment before surgery. Therefore, we conducted a propensity score matching analysis to balance these confounding factors (Table 2). Multivariable logistic regression analysis showed that preoperative Hb × SpO_2_ < aaHb was still significantly associated with the composite outcome in the matched cohorts (Table 3). It requires going back to the physiological function of hemoglobin. Preoperative Hb × SpO_2_ < aaHb indicates a decrease in blood oxygen content in cyanotic children. This may lead to inadequate tissue oxygen delivery for metabolic needs, consequently increasing the risk of postoperative organ dysfunction (7).

From the perspective of tissue oxygen delivery, preoperative Hb × SpO_2_ < aaHb may be a modifiable risk factor for cardiac surgery. It is estimated that the incidence of iron deficiency is up to 47.1% in children with cyanotic CHD (27). Despite the lack of high-level evidence, the consensus for patient blood management recommends early management anemia before cardiac surgery, including preoperative iron supplementation for iron-deficiency anemia and consideration of erythropoietin in patients with specific conditions (12, 28). According to the formula for calculating oxygen content (18), improvement of oxygen saturation may also be a strategy for selected children with cyanotic CHD, such as oxygen administration in concomitant parenchymal lung disease or deep cyanosis (29) and prostanoid pulmonary vasodilator use in severe pulmonary arterial hypertension (29, 30) and duct-dependent CHD (25, 31). However, further studies are required to determine whether these strategies can improve postoperative outcomes in cyanotic children with preoperative Hb × SpO_2_ < aaHb.

The authors acknowledge several limitations. First, there may have been selection bias in this single-institution retrospective study. Second, pulse oximetry performs poorly when SaO_2_ is <80% (32). The mean SpO_2_ was 4.6% higher than that of SaO_2_ in cyanotic children (32). In this study, we found that 100% was the optimal cutoff value of normal SpO_2_ (95–100%). Therefore, it was increased by approximately 5% on both sides of the equation (preoperative Hb × preoperative SpO_2_ = normal Hb × normal SpO_2_). To some extent, this counteracted the overestimation of oxygen saturation by SpO_2_ in cyanotic children. Meanwhile, multiple measurements of SpO_2_ are noninvasive, convenient and cost-effective. Moreover, an association, rather than causation, was identified between preoperative Hb × SpO_2_ < aaHb and postoperative outcomes. We could not eliminate the influence of other confounding factors such as socioeconomic status (33, 34) and chromosome abnormality (35), which were reported as prognostic factors for children with CHD but were not recorded for all children in our study.

## Conclusion

Our findings suggest that a preoperative Hb × SpO_2_ value below the lower limit of normal Hb is significantly associated with higher postoperative mortality and morbidity and is a potential criterion to evaluate preoperative anemia in children with cyanotic CHD. Prospective multicenter studies are required to confirm these findings.

## Data Availability Statement

The raw data supporting the conclusions of this article will be made available by the authors, without undue reservation.

## Ethics Statement

The studies involving human participants were reviewed and approved by the Ethics Committee on Biomedical Research, West China Hospital of Sichuan University (Reference Number, 2019-438). Written informed consent from the participants' legal guardian/next of kin was not required to participate in this study in accordance with the national legislation and the institutional requirements.

## Author Contributions

DZ, L-JD and Y-FL: contributed to the data curation, methodology, formal analysis, original draft, and final revision. M-LT: contributed to the conceptualization, methodology, interpretation, project administration, and final revision. All authors have read and approved the final manuscript.

## Funding

This work was supported by the West China Nursing Discipline Development Special Fund Project of Sichuan University (Grant Number, HXHL19061).

## Conflict of Interest

The authors declare that the research was conducted in the absence of any commercial or financial relationships that could be construed as a potential conflict of interest.

## Publisher's Note

All claims expressed in this article are solely those of the authors and do not necessarily represent those of their affiliated organizations, or those of the publisher, the editors and the reviewers. Any product that may be evaluated in this article, or claim that may be made by its manufacturer, is not guaranteed or endorsed by the publisher.

## Abbreviations

aaHb: age-adjusted lower limit of normal hemoglobin
CaO_2_: arterial oxygen content
CHD: congenital heart disease
*CI*: confidence interval
Hb: hemoglobin
*OR*: odds ratio
RACHS-1: the Risk Adjustment for Congenital Heart Surgery 1
SaO_2_: arterial oxygen saturation
SpO_2_: pulse oxygen saturation
VIF: variance inflation factor.
